# Proteomics-derived basal biomarker DNA-PKcs is associated with intrinsic subtype and long-term clinical outcomes in breast cancer

**DOI:** 10.1038/s41523-021-00320-x

**Published:** 2021-09-09

**Authors:** Karama Asleh, Nazia Riaz, Angela S. Cheng, Dongxia Gao, Samuel C. Y. Leung, Meenakshi Anurag, Torsten O. Nielsen

**Affiliations:** 1 Genetic Pathology Evaluation Centre, Department of Pathology and Laboratory Medicine, University of British Columbia, Vancouver, Canada; 2 Interdisciplinary Oncology Program, Faculty of Medicine, University of British Columbia, Vancouver, Canada; 3 Centre for Regenerative Medicine and Stem Cell Research, Aga Khan University, Karachi, Pakistan; 4 Baylor College of Medicine, Houston, TX USA

**Keywords:** Breast cancer, Prognostic markers

## Abstract

Precise biomarkers are needed to guide better diagnostics and therapeutics for basal-like breast cancer, for which DNA-dependent protein kinase catalytic subunit (DNA-PKcs) has been recently reported by the Clinical Proteomic Tumor Analysis Consortium as the most specific biomarker. We evaluated DNA-PKcs expression in clinically-annotated breast cancer tissue microarrays and correlated results with immune biomarkers (training set: *n* = 300; validation set: *n* = 2401). Following a pre-specified study design per REMARK criteria, we found that high expression of DNA-PKcs was significantly associated with stromal and CD8 + tumor infiltrating lymphocytes. Within the basal-like subtype, tumors with low DNA-PKcs and high tumor-infiltrating lymphocytes displayed the most favourable survival. DNA-PKcs expression by immunohistochemistry identified estrogen receptor-positive cases with a basal-like gene expression subtype. Non-silent mutations in *PRKDC* were significantly associated with poor outcomes. Integrating DNA-PKcs expression with validated immune biomarkers could guide patient selection for DNA-PKcs targeting strategies, DNA-damaging agents, and their combination with an immune-checkpoint blockade.

## Introduction

While gene expression profiling has refined breast cancer prognosis and helped guide treatment choices^1–3^, few advancements have been made in identifying practical biomarkers that can aid in tailoring treatments for the aggressive basal-like intrinsic subtype of breast cancer^4–6^.

The gene expression-defined basal-like breast cancer subtype is currently clinically approximated by triple-negative immunohistochemical (IHC) status, characterized by combined negativity for estrogen receptor (ER), progesterone receptor, and human epidermal growth factor receptor-2 (Her2). However, this IHC definition identifies a group with a heterogeneous biology^7–9^ that consists of at least four major molecular subgroups termed basal immune activated, basal immune suppressed, mesenchymal and luminal androgen receptor^10,11^. These subgroups have been repeatedly shown to differ in their clinical outcomes and exhibit a complex repertoire of somatic mutations, highlighting the complexity of guiding therapeutic choices for triple-negative breast cancers, including those with basal-like molecular biology^7,10^.

In an attempt to identify improved diagnostic tools and therapeutic options for this aggressive group of cancers, more precise basal biomarkers have been recently proposed based on new proteomic profiling data. A mass spectrometry-based analysis performed by the Clinical Proteomic Tumor Analysis Consortium group using fresh frozen materials from 122 TCGA breast cancer specimens reported DNA-dependent protein kinase catalytic subunit (DNA-PKcs) to be the most specific biomarker for the basal-like subtype^12,13^.

DNA-PKcs, encoded by *PRKDC*, is a member of the phosphatidylinositol 3-kinase–related family of protein kinases that plays a critical role in cell response to DNA damage and repair of double-strand breaks^14^. In response to DNA damage, the catalytic subunit of DNA-PKcs is recruited to the double-strand break site to bind to the Ku70/Ku80 heterodimer and form the DNA-PK serine/threonine-protein kinase complex^15^. This complex plays an important role in DNA damage response (DDR) and maintenance of genomic stability through the nonhomologous end-joining DNA repair pathway^16^. The binding of DNA-PKcs further phosphorylates and coordinates the activation of other proteins that mediate nonhomologous end-joining DNA repair^17,18^. Recently, DNA-PKcs has been proposed as an actionable therapeutic target for DNA damage in the breast and several other tumors types^19–22^ with DNA-PKcs inhibitors being actively assessed in clinical trials^14,23^. These findings along with recent evidence supporting the efficacy of DNA-PKcs in preclinical models support the development of DNA-PKcs targeting strategies in breast cancer^24^.

In the context of triple-negative breast cancer heterogeneity, *PRKDC* has been reported to be highly expressed in the basal immune activated and basal immune-suppressed molecular subgroups, while being depleted in the luminal androgen receptor and mesenchymal breast carcinomas^11^. DNA damage and double-strand break repair pathways have been shown to be specifically upregulated in basal-like breast cancers due to their high aberrant activation resulting from the DDR deficits, high mutational load, and genomic instability that characterize these tumors^8,25,26^.

In this study, we evaluated the prognostic capacity of the basal biomarker DNA-PKcs on a large tissue microarray series representing early-stage breast cancer patients. Following a prespecified study design, we tested the hypothesis that high expression of DNA-PKcs identifies cases with basal-like features and poor clinical outcomes. We further explored the value of combining DNA-PKcs IHC assessment with key immune biomarkers in the context of basal-like heterogeneity. In addition, we investigated the utility of DNA-PKcs as a basal biomarker in ER-positive breast cancers, correlating results with biological intrinsic gene expression subtype and genomic data.

## Results

### DNA-PKcs expression is associated with basal-like characteristics, adverse clinicopathological features and poor survival in the UBC series

**Fig. 1:**
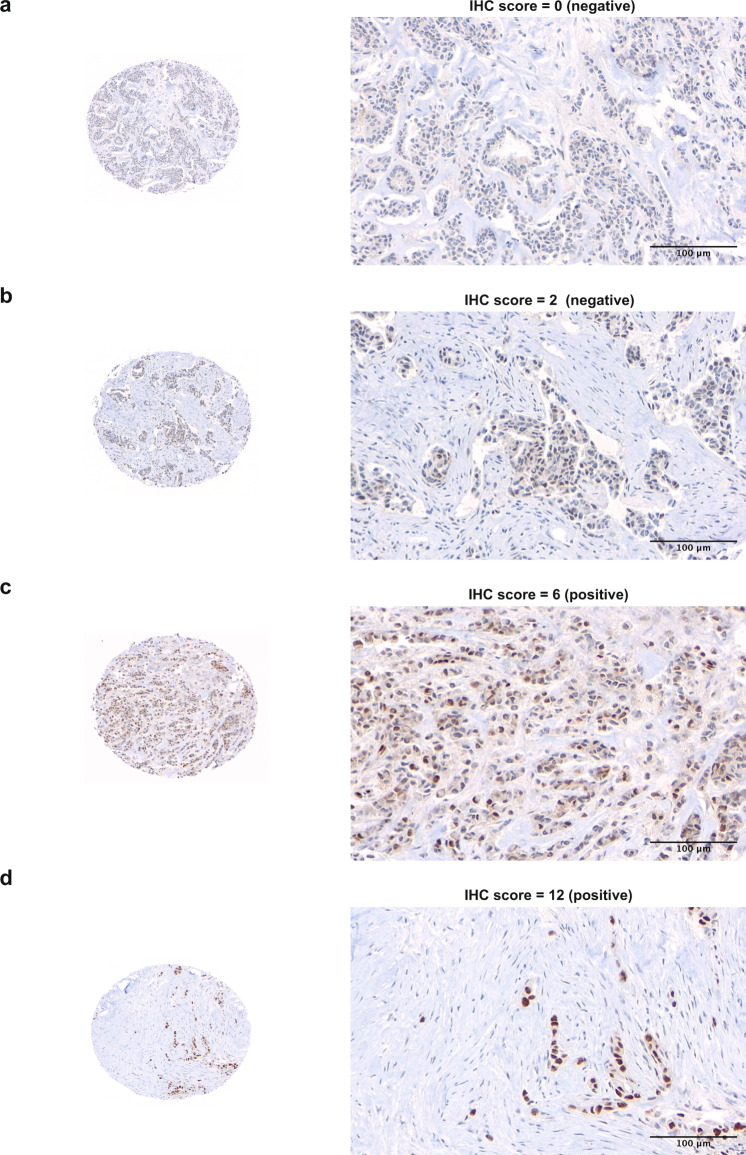
Representative images for nuclear expression of DNA-PKcs by immunohistochemistry. A case with negative staining for DNA-PKcs (IHC score = 0) is shown in (**a**), a case with 10% positivity and weak intensity (IHC score = 2) is shown in (**b**), a case with 40% positivity and moderate intensity (IHC score = 6) is shown in (**c**), and a case with 90% positivity and strong intensity (IHC score = 12) is shown in (**d**). The images were acquired at 20× objective magnification (200× original magnification) for the tissue microarray cores. Scale bar of 100 µm is shown. Abbreviations: IHC, immunohistochemistry.

**Fig. 2:**
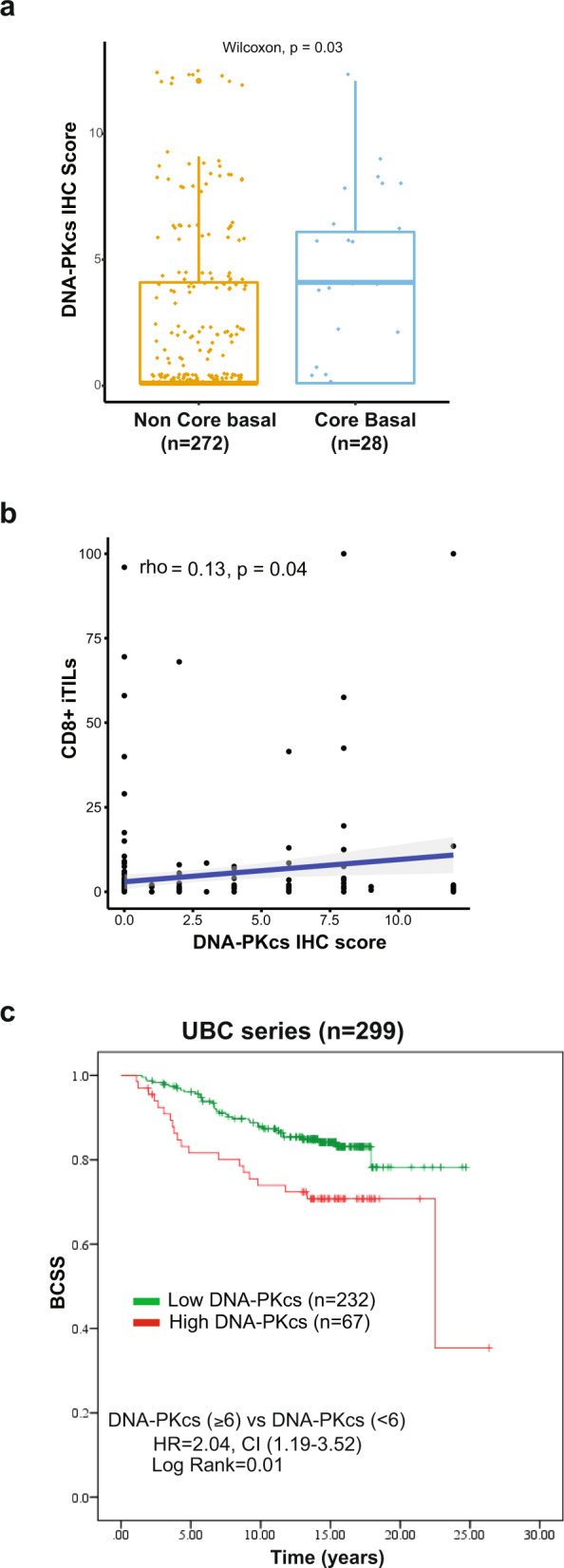
Analysis of DNA-PKcs expression in the UBC series (*n* = 300). **a** Boxplots showing expression levels of DNA-PKc by IHC according to “core basal status”. High expression of DNA-PKcs is associated with the core basal IHC phenotype (*p*-value < 0.05). Core basal subgroup is defined as ER-, PR-, Her2- and [EGFR + or CK5 + ]. The median (center bar), and the third and first quartiles (upper and lower edges, respectively) are shown. **b** Scatter plot of CD8 iTIL expression against DNA-PKcs IHC scores. Each data point represents one case. The Spearman correlation coefficient (rho) and p-values are displayed and indicate an overall weak but significant correlation. **c** Kaplan–Meier curves showing BCSS according to DNA-PKcs IHC expression status. Abbreviations: IHC immunohistochemistry, iTILs intraepithelial tumor-infiltrating lymphocytes, BCSS breast cancer-specific survival.

A total of 300 cases were evaluable for DNA-PKcs expression by immunohistochemistry (IHC) in the UBC series. Representative images of IHC expression of DNA-PKcs are displayed in Fig. 1. High expression of DNA-PKcs was found in 20.3% (*n* = 67) of cases and was associated with features of the aggressive disease including grade 3 histology, lymphovascular invasion, ER negativity, EGFR expression, CK5/6 expression, high proliferation index (Ki-67 ≥ 14%), and triple-negative status (Supplementary Table 1). DNA-PKcs expression was significantly higher in cases with the IHC core basal phenotype (defined as ER-negative, progesterone receptor-negative, Her2 negative, and [EGFR + or CK5 + ])^27^ compared to non-core basal cases (Fig. 2a and Supplementary Table 1). Additionally, DNA-PKcs expression was associated with high numbers of cytotoxic T-cells (CD8 + iTILs, Supplementary Table 1). When matching IHC expression data for DNA-PKcs with CD8 + iTILs on the same tissue core, a weak but significant correlation was observed (Fig. 2b).

The median follow-up for the UBC cohort was 12.7 years; tumors with high expression of DNA-PKcs were found to be significantly associated with lower breast cancer-specific survival (HR 2.04, 95% CI 1.19–3.52, *p* = 0.01) when compared to cases with low DNA-PKcs (Fig. 2c).

### Validation of the prognostic significance of DNA-PKcs in the BC Cancer series

Observations from the UBC series cohort were next validated in the larger, independent BC Cancer series cohort (Supplementary Data 1) wherein the mean age at diagnosis was 58.9 years and the median duration of follow-up was 12.5 years (Table 1).

**Table 1:** Association of DNA-PKcs expression with clinicopathological features in the British Columbia Cancer series.

Clinicopathological features	DNA-PKcs expression (IHC score)		*p*-value
	Low (<6) *n* = 1783 (%)	High (≥6) *n* = 618 (%)	
***Age at diagnosis (years)***			
<50	553 (31.0)	217 (35.1)	0.06
≥50	1230 (69.0)	401 (64.9)	
***Tumor size (cm)***			
≤2	915 (51.6)	301 (48.9)	0.26
>2	859 (48.4)	314 (51.1)	
***Tumor grade***			
1 & 2	783 (45.9)	242 (40.4)	0.02
3	922 (54.1)	357 (59.6)	
***Axillary lymph node status***			
Negative	994 (55.9)	337 (54.6)	0.59
Positive	785 (44.1)	280 (45.4)	
***Ki-67 expression***			
<14%	919 (55.3)	264 (45.8)	<0.001
≥14%	743 (44.7)	313 (54.2)	
***Lymphovascular invasion***			
Negative	938 (54.9)	303 (50.6)	0.07
Positive	770 (45.1)	296 (49.4)	
***ER expression***			
Negative	397 (22.3)	280 (45.3)	<0.001
Positive	1381 (77.7)	338 (54.7)	
***Progesterone receptor expression***			
Negative	771 (45.7)	326 (55)	<0.001
Positive	917 (54.3)	267 (45)	
***HER2 expression***			
Negative	1555 (88.9)	470 (78.1)	<0.001
Positive	195 (11.1)	132 (21.9)	
***EGFR expression***			
Negative	1480 (90.2)	410 (74.3)	<0.001
Positive	161 (9.8)	142 (25.7)	
***CK5/6 expression***			
Negative	1487 (93)	472 (84.3)	<0.001
Positive	112 (7)	88 (15.7)	
***Breast cancer subtypes***			
Luminal A ([ER + or PR + ], HER2-, low Ki67)	786 (47.4)	179 (31)	<0.001
Luminal B ([ER + or PR + ], HER2-, high Ki67)	456 (27.5)	111 (19.3)	
Luminal B ([ER + or PR + ], HER2 + )	102 (6.1)	44 (7.6)	
ER−, PR−, HER2 +	89 (5.4)	83 (14.4)	
ER−, PR−, HER2−	226 (13.6)	160 (27.7)	
***Core basal subtype***			
Yes	121 (7.9)	117 (20.3)	<0.001
No	1417 (92.1)	460 (79.7)	
***Stromal TILs (H&E)***			
<10%	1401 (83.7)	431 (75.7)	<0.001
≥10%	272 (16.3)	138 (24.3)	
***CD8 iTIL Count***			
<1	1163 (67.6)	352 (59.4)	<0.001
≥1	559 (32.5)	241 (40.6)	

Abbreviations: *IHC* immunohistochemistry, *ER* estrogen receptor, *PR* progesterone receptor, *HER2* human epidermal growth factor receptor 2, *EGFR* epidermal growth factor receptor, *CK* cytokeratin, *H&E* hematoxylin and eosin, *CD* cluster of differentiation.

**Fig. 3:**
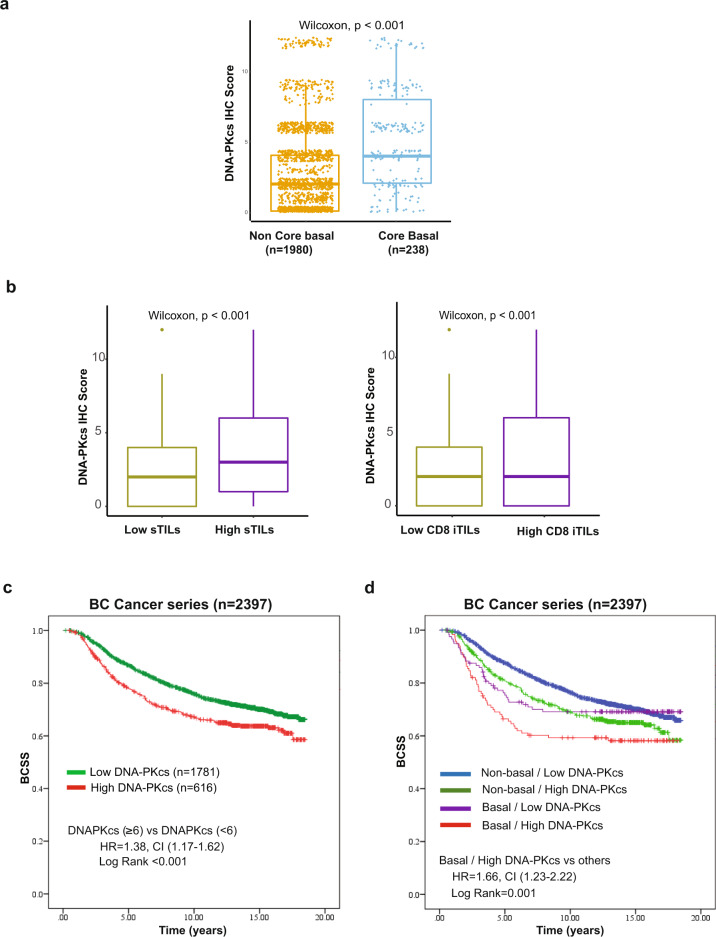
Analysis of DNA-PKcs expression in the BC Cancer series (*n* = 2401). **a** Boxplots showing the expression levels of DNA-PKcs by IHC according to “core basal status”. High expression of DNA-PKcs is associated with the core basal IHC phenotype (*p*-value < 0.001). Core basal is defined as ER-, PR-, Her2- and [EGFR + or CK5 + ]. The median (center bar), and the third and first quartiles (upper and lower edges, respectively) are shown. **b** Boxplots showing the expression levels of DNA-PKcs by IHC according to “sTILs” and “CD8 + iTILs” categories. High expression of DNA-PKcs is associated with high expression of sTILs and high expression of CD8 + iTILs. The median (center bar), and the third and first quartiles (upper and lower edges, respectively) are shown. **c** Kaplan–Meier curves for BCSS in the BC Cancer series according to DNA-PKcs IHC expression status. **d** Kaplan–Meier curves for BCSS stratified according to core basal status and DNA-PKcs IHC expression. Core basal tumors with high DNA-PKcs expression are associated with the worst BCSS. Abbreviations: IHC immunohistochemistry, sTILs stromal tumor-infiltrating lymphocytes, iTILs intraepithelial tumor-infiltrating lymphocytes, BCSS breast cancer-specific survival.

Of the primary tumor samples, 2401 were interpretable for DNA-PKcs immunostaining. The original version of the BC Cancer series TMA had 3992 cases^28,29^, but since that time many cores have been cut through and source blocks exhausted, such that interpretable data for the current study could be generated for 2401 cases. Among these, high expression of DNA-PKcs was observed in 25.7% (618/2401 cases) (Table 1). A significant association was observed between tumors with high expression of DNA-PKcs and adverse pathological features including grade 3 histology, high Ki-67 proliferation index (defined as ≥14%), hormone receptor negativity, Her2 positivity, expression of basal biomarkers including CK5/6, EGFR, and a triple-negative phenotype (*p* < 0.001) (Table 1). In addition, DNA-PKcs expression was found to be significantly associated with core basal tumors (*p* < 0.001) (Fig. 3a) (Table 1). Using prespecified criteria and the scoring methodology as published by others^30^, we analyzed the correlation of DNA-PKcs expression with infiltrating lymphocytes (stromal H&E sTILs and CD8 + iTILs). We found that tumors categorized with high DNA-PKcs expression were highly significantly associated with the expression of these immune biomarkers (*p* < 0.001) (Table 1). Further assessment of DNA-PKcs expression revealed significantly higher scores in cases characterized by high H&E sTILs and CD8 + iTILs (Fig. 3b).

We next evaluated the prognostic significance of DNA-PKcs expression in the BC Cancer series, and confirmed that cases with high DNA-PKcs expression are associated with poor BCSS (HR 1.38, 95% CI 1.17–1.62; *p* < 0.001) (Fig. 3c). Upon stratification by ER status, high DNA-PKcs expression retained a significant association with poor BCSS in ER- tumors (HR 1.44, 95% CI 1.12–1.87; *p* = 0.005) compared to ER + tumors (HR 1.19, 95% CI 0.95–1.48; *p* = 0.13) (Supplementary Fig. 1a–1b).

Next, we performed multivariate analysis using Cox proportional hazards model to assess the independent prognostic relevance of DNA-PKcs expression adjusted for clinicopathological variables (age, tumor size, histological grade, axillary lymph node status), breast cancer subtypes, and systemic treatments. High expression of DNA-PKcs remained an independent prognostic factor indicative of poor BCSS (HR 1.33, 95% CI 1.10–1.60; *p* = 0.002) (Table 2).

**Table 2:** Multivariate analysis for DNA-PKcs expression in the British Columbia Cancer series including clinicopathological features, treatment, and subtype information.

Covariates in the model	Breast cancer specific survival	
	Adjusted HR (95% CI)	*p*-value
***Age at diagnosis***		
<50	1	0.60
≥50	1.07 (0.85–1.36)	
***Tumor size (cm)***		
≤2	1	<0.001
>2	1.53 (1.29–1.81)	
***Tumor grade***		
1 & 2	1	<0.001
3	1.58 (1.30–1.88)	
***Axillary lymph node status***		
Negative	1	<0.001
Positive	2.50 (2.00–3.14)	
***Breast cancer subtypes***		
Luminal A	1	<0.001
Luminal B/Ki67+	1.72 (1.40–2.11)	
Luminal B/ HER2+	1.78 (1.32–2.41)	
HER2+	1.70 (1.26–2.28)	
Basal	1.55 (1.17–2.06)	
***Adjuvant systemic treatment***		
Tamoxifen only	0.82 (0.63–1.06)	0.45
Chemotherapy only	0.84 (0.62–1.15)	
Tamoxifen + chemotherapy	0.80 (0.55–1.17)	
***DNA-PKcs IHC expression***		
Low (<6)	1	0.002
High (≥6)	1.33 (1.10–1.60)	

HR and 95% CI are derived from multivariable analysis adjusted for age at diagnosis, tumor size,

tumor grade, nodal status, breast cancer subtypes, and adjuvant systemic treatments using the Cox regression model. Abbreviations: *IHC* immunohistochemistry, *HR* hazard ratio, *CI* confidence interval.

Breast cancer subtypes: Luminal A, ER + or PR + and Ki-67 < 14%; Luminal B/Ki67 + , ER + or PR + and Ki-67 ≥ 14%; Luminal B/HER2 + , ER + or PR + and HER2 + by IHC or FISH; HER2 + , ER- PR- and HER2 + by IHC or FISH; Basal, triple-negative and EGFR + or CK5 + .

### Prognostic stratification of basal cases based on the combination of DNA-PKcs and immune biomarkers

The core basal phenotype defined by a 5-biomarker immunopanel (ER-negative, progesterone receptor-negative, Her2 negative, and [EGFR + or CK5 + ]) has been previously shown to more specifically identify cases with the basal-like gene expression subtype^27^ and to provide superior prognostic information when compared to an IHC definition that is based simply on triple-negative expression for the estrogen, progesterone and Her2 receptors^31^. Thus, we specifically examined the prognostic significance of DNA-PKcs expression in the IHC based core basal (vs non-core basal) subtype and found that tumors characterized by both high expression DNA-PKcs and by a core basal phenotype displayed the worst BCSS (HR 1.66, 95% CI 1.23–2.22; *p* = 0.001) compared to other groups (Fig. 3d).

**Fig. 4:**
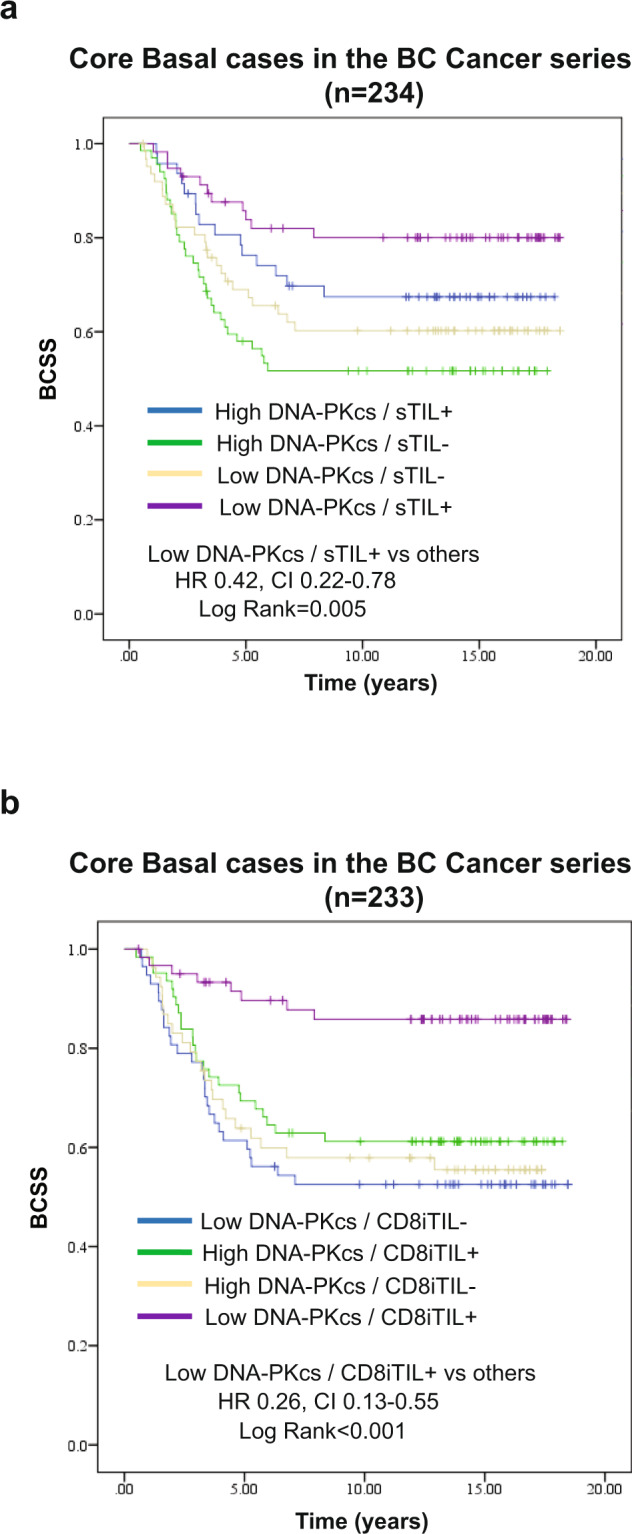
Prognostic stratification of core basal cases by DNA-PKcs and immune biomarkers in the BC Cancer series. **a** Within core basal tumors, the presence of sTILs in combination with low DNA-PKcs expression is associated with better BCSS. **b** Within core basal tumors, the presence of CD8 + iTILs in combination with low DNA-PKcs expression is associated with better BCSS. Abbreviations: IHC immunohistochemistry, BCSS breast cancer-specific survival, sTILs stromal tumor-infiltrating lymphocytes, iTILs intraepithelial tumor-infiltrating lymphocytes.

Given that *PRKDC*, the gene encoding for DNA-PKcs, is characteristic of both the basal immune activated and basal immune-suppressed RNA-based subgroups of triple-negative breast cancer^11^, we investigated the prognostic significance of the combination of key immune biomarkers (sTILs and CD8 + iTILs) and DNA-PKcs expression status within the core basal subtype. We found that low DNA-PKcs expression concurrent with the presence of stromal TILs correlated with superior survival in the core basal tumors (HR 0.42, 95% CI 0.22–0.78; *p* = 0.005) (Fig. 4a). Similar results were observed when we used ≥30% as the cutpoint for defining high levels of H&E sTILs, a value used by others in recently published studies^32,33^ (Supplementary Fig. 2).

The cytotoxic T-cell subset showed an even more marked association with good prognosis: cases with low DNA-PKcs that had high levels of CD8 + iTILs were associated with a significantly better BCSS (HR 0.26, 95% CI 0.13–0.55; *p* < 0.001) (Fig. 4b), defining a group of patients with disease-specific survival better than 80% even 15 years after being diagnosed with triple-negative breast cancer.

### DNA-PKcs and mRNA *PRKDC* expression are associated with PAM50 intrinsic subtype and poor clinical outcomes

**Fig. 5:**
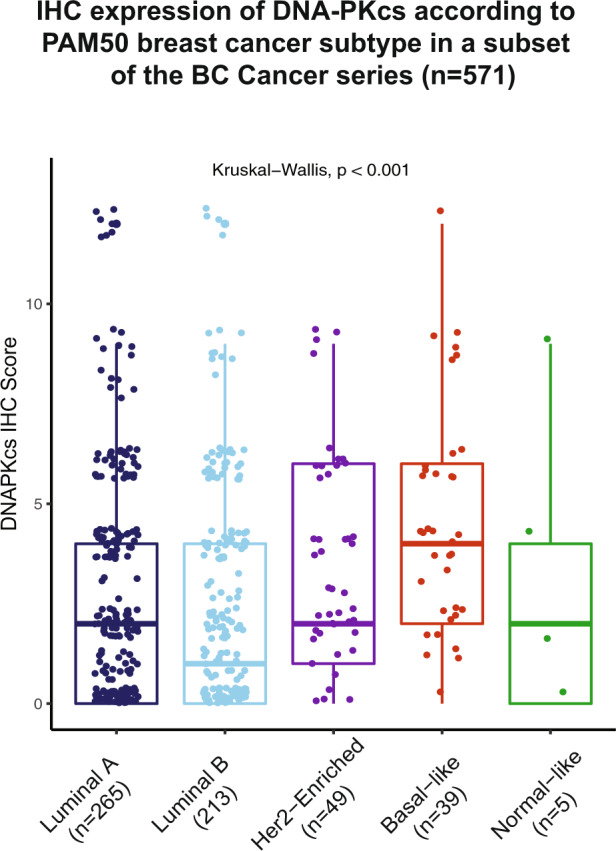
Association of DNA-PKcs expression with PAM50 intrinsic subtype in a subset of the BC Cancer series. Boxplots showing the expression levels of DNA-PKcs by IHC across the different PAM50 gene expression intrinsic subtypes. High expression of DNA-PKcs is associated with basal-like PAM50 subtype (*p*-value < 0.001). The median (center bar), and the third and first quartiles (upper and lower edges, respectively) are shown.

To date, successful basal biomarkers that have been validated against gold-standard gene expression assays are mostly limited to the triple-negative breast cancer setting^31^ with very few applicable in the context of ER positivity^34,35^. However, there is a proven subset of basal-like gene expression in the literature that is ER positive^36–38^. Thus, we aimed to assess the value of DNA-PKcs as a basal marker on datasets with gene expression profile data that include ER-positive cases. We tested the association between DNA-PKcs IHC expression and PAM50 intrinsic subtype on a set of 825 cases in the BC Cancer series previously profiled by quantitative reverse transcription-polymerase chain reaction for PAM50 gene expression^39^. The majority of these cases corresponded to clinically ER + patients that were treated with adjuvant tamoxifen^39^; a total of 571 had available data for both mRNA PAM50 intrinsic subtype and DNA-PKcs expression by IHC. Basal-like PAM50 tumors were characterized by higher IHC scores for DNA-PKcs expression when compared to the other PAM50 subtypes (*p*-value<0.001) (Fig. 5).

**Fig. 6:**
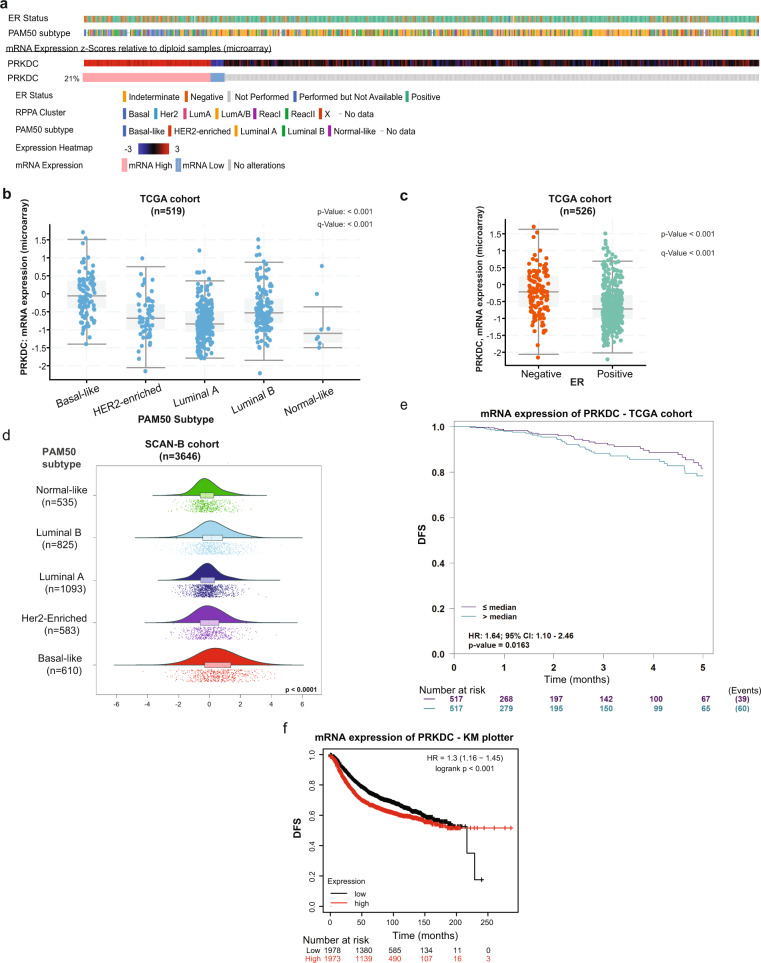
Analysis of *PRKDC* expression using publicly-available breast cancer datasets. **a** Oncoprint outlining the biological classifications of 825 cases included in the TCGA invasive breast cancer cohort according to ER status, PAM50 subtype, RPPA cluster, and *PRKDC* mRNA expression as determined by microarray. **b**–**c** Boxplots showing the expression levels of *PRKDC*, as derived from microarray in the TCGA invasive breast cancer cohort, is significantly associated with basal-like PAM50 intrinsic subtype. The median (center bar), and the third and first quartiles (upper and lower edges, respectively) are shown. **c** Data were obtained through the cBioPortal for Cancer Genomics database^74^. **d** Raincloud plots showing the expression level of PRKDC, as derived from RNA-seq on the SCAN-B breast cancer cohort, is significantly associated with basal-like PAM50 intrinsic subtype. **e**–**f** Kaplan–Meier survival curves showing the association between *PRKDC* expression and DFS on cases from the TCGA invasive breast cancer cohort (**e**) and 35 Gene Expression Omnibus breast cancer datasets (**f**). Plots were generated using the bc-GenExMiner v4.5^75^ and the KMplotter analysis platform curated from 35 Gene Expression Omnibus breast cancer datasets^41^. Abbreviations: ER estrogen receptor, IHC immunohistochemistry, DFS disease-free survival.

We next assessed the expression of the DNA-PKcs gene (*PRKDC*) at the transcriptomic level using data from the TCGA invasive breast cancer cohort^8^ (Fig. 6a). Higher *PRKDC* expression is significantly associated with basal-like PAM50 subtype and ER negativity (Fig. 6b, c). In addition, high *PRKDC* expression is also associated with basal-like gene signature within ER + tumors in the TCGA cohort (Supplementary Fig. 3). We further validated the association between *PRKDC* expression and the basal-like PAM50 subtype using data obtained from a contemporary collection of primary breast cancer tissues from women enrolled in the SCAN-B trial^40^ (NCT02306096) (Fig. 6d). High *PRKDC* was significantly associated with poor disease-free survival rates in the TCGA cohort (Fig. 6e) and when applying KMplotter to 35 publicly available Gene Expression Omnibus datasets^41^ (Fig. 6f). Taken together, our results show that both DNA-PKcs protein and *PRKDC* transcript are biomarkers that help identify basal-like cases both within ER + and ER- breast cancers.

### *PRKDC* non-silent somatic mutations are associated with poor clinical outcomes

**Fig. 7:**
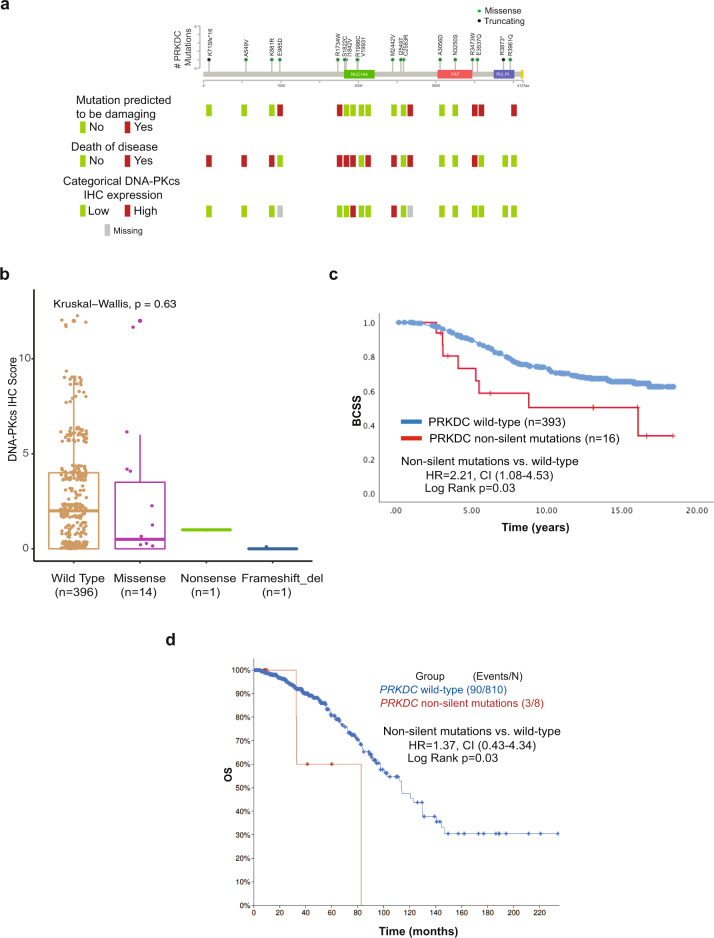
*PRKDC* non-silent somatic mutations are associated with poor clinical outcomes. **a** Lollipop plot for *PRKDC* mutations identified in a set of 640 tamoxifen-treated, clinically ER + patients. Green-filled circles denote missense mutation and black-filled circles denote truncating mutations. Protein domains are indicated as follows: green, NUC194 domain B in the catalytic subunit of DNA-dependent protein kinases; red, FAT domain present in the PIK-related kinases; dark-blue, Phosphoinositide 3-kinase (PI3K) domain. A complete description of *PRKDC* mutations has been previously published and can be found in Supplementary data 3 of Griffith et al.^42^. Mutation pathogenicity, clinical annotation of death due to disease, and DNA-PKcs expression categories by IHC for the corresponding cases are displayed. **b** Boxplots showing the expression levels of DNA-PKcs by IHC according to mutation status and its type. The median (center bar), and the third and first quartiles (upper and lower edges, respectively) are shown. **c** Kaplan–Meier curve for BCSS in the subgroup of ER + cases treated with tamoxifen in the BC Cancer series according to *PRKDC* mutation status (*n* = 409). **d** Kaplan–Meier curves for OS in the TCGA cohort of invasive breast cancer according to *PRKDC* mutation status (*n* = 818). Abbreviations: ER estrogen receptor, BCSS breast cancer-specific survival, OS overall survival, IHC immunohistochemistry.

We next aimed to correlate our findings with somatic mutations in the *PRKDC* gene encoding for DNA-PKcs. The somatic mutations previously published from a subset of 640 tamoxifen-treated, clinically ER + patients from the BC Cancer series were used in this analysis^42^ (Supplementary Data 1). Among those cases, 420 cases also had data for DNA-PKcs expression by IHC generated for the current study, for which 16 had non-silent and 8 had silent mutations in *PRKDC* whereas 396 were wild type. The majority of non-silent mutations were missense (14/16), with 1 additional nonsense and 1 frameshift (Fig. 7a). Four of the 14 missense mutations with available data for IHC DNA-PKcs expression were predicted to be damaging to the protein function using the “Mutation Assessor” tool (Supplementary Data 1). When testing the association between mutation status and DNA-PKcs expression by IHC, the two cases with truncating mutations were negative for IHC expression (Fig. 7a). In addition, the majority of cases with missense mutations displayed low expression for DNA-PKcs by IHC. A comparison of DNA-PKcs IHC expression between wild type vs. non-silent mutated cases was insufficiently powered to observe a significant association due to the small number of mutated cases (Fig. 7b).

We further investigated the prognostic implications of *PRKDC* somatic mutations in this cohort and found that cases classified as having non-silent mutations in *PRKDC* exhibited significantly poor clinical outcomes when compared to cases characterized with wild-type *PRKDC* (HR 2.21, 95% CI 1.08–4.53; *p* = 0.03) (Fig. 7c). The TCGA dataset showed non-silent somatic mutations in *PRKDC* in 8 of 818 cases, which despite limited power showed a similarly significant adverse prognostic association (Fig. 7d). Amongst ER-negative cases, only 2 of 179 in the TCGA cohort and 6 of 233 in the SCAN-B cohort had PRKDC somatic mutations, numbers too small for meaningful survival analyses.

## Discussion

In this study, we evaluated the prognostic capacity of a basal subtype biomarker, DNA-PKcs, derived from a published high-quality comprehensive proteomic profiling study on TCGA breast cancer samples^12^. Following prespecified study design and methodology adhering to REMARK criteria on both training and validation cohorts^43^, we demonstrate, using large cohorts of clinically-annotated breast cancer cases, that IHC expression of DNA-PKcs is associated with the basal-like subtype, high-risk clinicopathological factors, and poor prognosis. These findings are consistent with previous reports showing that high expression of *PRKDC* is associated with poor clinicopathological features and clinical outcomes in breast cancer at the transcriptomic level^19,44^.

The association of high expression of DNA-PKcs with poor clinical outcomes in our cohort was more evident within ER- cases when compared to ER + . These findings might be explained by preclinical studies showing that ER signaling regulates DNA damage response targets including DNA-PKcs and ATM, with the majority of ER + tumors displaying relatively low protein expression of DNA-PKcs^45^. In addition, DNA damage processes are particularly characteristic of basal tumors, when compared to ER + tumors that have less genomic instability^8,25,26^, thus consistent with our observation of low overall protein expression of DNA-PKcs within the majority of ER + when compared to ER− breast cancers. Interestingly, a dual role for DNA-PKcs has been further suggested in preclinical models, as a tumor suppressor in premalignant stages maintaining genome integrity; while in an aggressive and advanced stage, DNA-PKcs could indicate high genomic instability, thus acting as an oncogenic driver^23^.

A previous study by the Nottingham breast cancer group reported that IHC expression of DNA-PKcs was significantly associated with good clinical outcomes in breast cancer^46^. These observations were mainly seen in the ER + subgroup and as the authors noted, are contradictory to the preponderance of the pre-clinical literature showing that DNA-PKcs phosphorylates and stabilizes ER and hence that low levels of DNA-PKcs would be expected to contribute to a reduced ER signaling resulting in less aggressive ER + tumors^47–50^. Furthermore, the authors noted the discordance of their outcome associations from those reported in other transcriptomic studies in breast cancer^19,44^ and several other tumors^20,21,23,51^. The apparent discordance with our current study and other transcriptomic studies^19–21,23,44,51^ might be because the Nottingham study applied data-driven cutpoints to maximize outcome differences in their data set, in contrast to our study that applied a prespecified externally-validated cutpoint on first a breast cancer training and then on a larger independent validation set. The discordant findings might also be explained by the complexity of DNA-PKcs expression due to changes in protein post-translational modifications that are involved in the DNA damage repair process^52–54^ and could affect the role of ER signaling in regulating DDR targets including DNA-PKcs^45^.

In our study, we demonstrated the capacity of DNA-PKcs as a basal biomarker that is applicable even in the setting of ER positivity, validated its association with the basal-like PAM50 subtype and correlated results with *PRKDC* mutational status in a large subset of ER + cases. Within the clinically ER + group, cases with low DNA-PKcs expression were luminal by PAM50 gene expression while those with a high DNA-PKcs profiled as basal-like.

Profiling of the ER + cases for mutations in the *PRKDC* gene further showed that non-silent mutations correlated with poor survival. Interestingly, the original study^42^ that performed targeted sequencing on the ER + subset of cases included in this study reported non-silent somatic mutations in *PRKDC* to be one of the topmost significant poor outcome drivers in ER + cases. These mutations have been further reported to be associated with downregulated *ATM* levels^55^, potentially driving resistance to endocrine therapy^42^.

In support of our findings, mutations in *PRKDC* have been previously implicated in breast cancer initiation and progression^44,56^. In our study, the majority of non-silent mutations (including both missense and truncating) resulted in a lower DNA-PKcs protein expression. The majority of *PRKDC* missense mutations we identified would result in either impaired function or a lower expression of the DNA-PKcs protein. While only a third of missense mutations were predicted to be damaging, the majority of other “likely benign” mutated cases still correlated with poor disease-specific survival. These findings suggest that these mutated cases, being defective for DNA damage repair, are impaired in their capacity to maintain genomic stability and consequently evolve to behave aggressively. *PRKDC* mutant breast tumors (including those bearing loss-of-function mutations) are characterized by high mutational load and genomic instability^56^, suggesting that these tumors should correlate with poor prognosis regardless of DNA-PKcs protein levels. Since the fraction of mutated *PRKDC* tumors in breast cancer is very low (1%), our main findings reporting that low expression of DNA-PKcs correlates with good survival is driven by wild-type tumors. In this context, DNK-PKcs IHC expression and *PRKDC* mutation should be considered in combination to define a specific subgroup with better prognostication.

In relation to treatment, the expression of DNA-PKcs has been reported to drive resistance to chemotherapy and radiotherapy in preclinical models^19,57^, whereas inhibition of DNA-PKcs has been shown to sensitize breast cancer cells to these treatment modalities^22,24^.

DNA-PKcs is a key regulator for maintaining genomic integrity, cell cycle, and DNA repair through forming complexes with Ku70/80 to mediate DDR^16,18^. However, its aberrant high expression in tumors could be indicative of inherent DDR deficits and high genomic instability that drive the resistance of these tumors to chemoradiotherapy. High DNA-PKcs levels have also been shown to be induced after chemotherapy or radiotherapy treatments in a manner not dependent on endogenous levels of DNA damage, but rather on drug-induced levels precipitated by damaging agents^57–60^. Thus, the prognostic and predictive capacity of DNA-PKcs may well best be assessed on specimens taken from primary tumors prior to systemic chemotherapy and/or metastatic disease.

In the context of basal-like breast cancer heterogeneity, our study shows that tumors exhibiting the core basal-like phenotype had a higher expression of DNA-PKcs when compared to tumors characterized as non-core basal. Furthermore, we found a significant association between high DNA-PKcs and high numbers of TILs and cytotoxic CD8 + lymphocytes. These findings might be explained by the high genomic instability that characterizes basal tumors with high DNA-PKcs expression, resulting in an accumulation of genetic alterations and a consequent high mutational load^61^ that could lead to neoantigen production, inducing an immunogenic antitumor response (basal-like immune hot phenotype). While the process of neoantigen production is an established consequence of genomic alterations, the immunogenicity of these tumors is highly dependent on the preexisting inflammatory milieu of the host^62^ and thus the basal subset exhibiting high expression for DNA-PKcs, but low expression of sTILs and CD8 iTILs, represents a “basal immune cold” subset. This basal subset exhibited the worst survival in our study.

To date, the major success made in treating triple-negative breast cancer patients, with PARP inhibitors targeting the homologous recombination pathway, has been mainly limited to a small fraction of patients who harbor *BRCA* mutations or homologous recombination pathway defects^4,63,64^. However, limited data exist on the role of PARP inhibitors across the diverse subsets of triple-negative, basal-like and sporadic homologous deficient breast cancers^64,65^.

DNA-PKcs, as one of the key proteins involved in the DDR, could aid in matching basal patients to DNA-PKcs targeting strategies, DNA-damaging agents, or PARP inhibitors^24^. Specifically, since PARP inhibitors induce the nonhomologous end-joining process among homologous recombination deficient tumors, DNA-PKcs could represent a promising therapeutic target in this particular setting^66^. Furthermore, with the contribution of the DNA damage process to high mutational load and antigenicity^67^, neoantigen production could be further increased as a result of mutations induced by DNA damaging agents. It has been shown that in response to DNA damaging chemotherapy, DDR can promote signalling pathways resulting in a release of proinflammatory cytokines including type I interferon and nuclear factor-kB^68^.

Our study has several limitations. While our primary hypothesis has been tested on large cohorts of patients following a prespecified design and scoring methodology, yielding powered positive results, future studies using samples from clinical trials are critical to establishing the capacity of DNA-PKcs as a biomarker to predict benefit from DNA damaging agents, PARP inhibitors and/or immunotherapies among basal breast cancer patients. Additionally, the prognostic capacity of DNA-PKcs in the context of basal immune heterogeneity was based on evaluating sTILs and CD8 + iTILs in these tumors. Given the contribution of many cell populations, protein components, and their cross-talk to form an effective anti-tumor immune response, the enumeration of cytotoxic T cells in tumors is insufficient to characterize complex immune distinctions. Furthermore, our study included pretreatment specimens from early-stage breast cancer patients; DNA-PKcs expression could be upregulated after exposure to chemotherapy or radiotherapy during subsequent tumor progression. Thus, the prognostic and predictive capacity of DNA-PKcs expression should be further evaluated after exposure to chemoradiation particularly amongst basal patients who progress to metastatic disease.

In conclusion, this study demonstrates the prognostic capacity of DNA-PKcs, a basal breast cancer biomarker derived from comprehensive proteomic profiling of breast cancer. The integration of DNA-PKcs expression along with established immune biomarkers stratifies major risk differences within the basal-like subtype. Such findings, when applied on clinical trial series would aid in matching basal patients to DNA-PKcs targeting strategies, DNA-damaging agents, and their combination with immune checkpoint blockade.

## Methods

### Study cohorts

Two independent, well-annotated cohorts corresponding to patients diagnosed with stage I–III breast cancer were included in the current study. The staining protocol, scoring criteria, and clinical data analysis were first evaluated on a set of female patients diagnosed with invasive breast cancer (*n* = 330) at the University of British Columbia (UBC) hospital between 1998–2002, designated as the UBC series. The second cohort was used for subsequent detailed analyses and is comprised of primary invasive breast cancer cases diagnosed in the province of British Columbia at the British Columbia Cancer Agency between 1986–1992, referred to as the BC Cancer series. These patients were treated in accordance with the provincial guidelines during the specified time period. The characteristics of these cohorts have been described previously^28,29^.

Patients diagnosed with ductal carcinoma in situ only, metastatic disease at presentation, and those who received neoadjuvant therapies were excluded.

### Ethics approval and study design

This study was approved by the research ethics board of UBC and the BC Cancer Breast Cancer Outcomes unit (approval number: H17-01207). The current hypothesis-based retrospective biomarker study was conducted in accordance with the Reporting Recommendations for Tumor Marker Prognostic Studies (REMARK) guidelines^43^. Prespecified assessment criteria were used for IHC scoring of the biomarkers of interest. Potential hypotheses were initially tested in the UBC series, with the independent BC Cancer series used for subsequent validation studies following a formal prespecified analysis plan approved at a meeting of the Breast Cancer Outcomes Unit at BC Cancer. Consent for the use of previously assembled patient specimens was obtained under a waiver of informed consent policy without identification of patient information.

### Tissue microarrays and immunohistochemistry

Formalin-fixed paraffin-embedded tumor blocks of primary surgical specimens were used to construct a series of 0.6 mm core tissue microarrays (TMAs) for both study cohorts as described previously^69^. For the UBC series, duplicate 0.6 mm cores were extracted from each pathology block and embedded into three TMA recipient blocks, while seventeen TMA blocks needed to be constructed to represent the BC Cancer series (1 core per patient)^69^. Serial 4μm sections from these TMAs were previously stained for the following IHC biomarkers included in this study: ER, progesterone receptor, HER2, Ki-67, cytokeratin (CK5/6), and EGFR. The detailed protocols for IHC staining, scoring criteria of these biomarkers, and definitions of IHC-based breast cancer subtypes have been described previously^31^. Stromal tumor-infiltrating lymphocytes were assessed as per recommendations of the International TIL Working Group^70^. To assess the suitability of TMAs for assessing stromal tumor-infiltrating lymphocytes (sTILs), we scored sTILs on digitized full-face hematoxylin and eosin (H&E) stained sections and corresponding 0.6 mm TMA cores from 317 cases from the BC Cancer series. A good correlation (spearman *rho* = 0.67) was observed (Supplementary Fig. 4). Hence for further analyses TMAs were utilized with a 10% cutoff as described previously^71^. In addition, TMA sections were scored for CD8 ^+^ TILs in the intraepithelial compartment (iTILs) using established, analytically validated IHC staining and interpretation methods as previously published by our group^72,73^. We chose to analyze CD8 ^+^ iTILs based on previous observations that this biomarker could define the relevant subset of cytotoxic immune cells that drives anti-tumor immune response and associates with good prognosis^72^. Array sections at 4 µm were mounted on glass slides and baked for an hour at 60 °C to prepare for staining on a Ventana Discovery XT automated stainer (Ventana Medical Systems, Tucson, AZ). Antigen retrieval was performed using Cell Conditioning 1 antigen retrieval (Ventana Medical Systems) followed by 2 h of primary antibody incubation at room temperature, and detected using a ChromoMap DAB Detection Kit (Ventana Medical Systems). IHC staining of DNA-PKcs was performed with anti DNA-PKcs rabbit monoclonal primary antibody (clone Y393, dilution 1:500, Abcam, cat# ab32566). Slides were then incubated with a secondary antibody (UltraMap anti-Rb HRP) for an additional 16 min. Separate TMAs that included normal breast, breast, and ovarian cancer tissues were used as positive controls. The stained TMA slides were digitally scanned and DNA-PKcs nuclear expression in the tumor cells was visually scored by a pathologist blinded to clinical data.

Scoring of DNA-PKcs was performed following published criteria previously employed by other groups, using an IHC scoring system based on the proportion and intensity of nuclear staining observed in the invasive carcinoma cells^30^. The positivity proportion scores were captured as a continuous variable for each core and then categorized into four scores as follows: 0, no positive tumor cells; 1, <10%; 2, 10–34%; 3, 35–74%, and 4, ≥75%. The staining intensity was reported as weak (1), moderate (2), or strong (3). The IHC score was computed by multiplying the proportion of positive cells (categorized as 1–4) by the intensity score. This computed IHC score ranged from 0–12 and was used for the final scoring of DNA-PKcs by IHC. For cases with duplicate cores, the higher IHC score was used for analysis. Low expression of DNA-PKcs was defined as an IHC score of <6 whereas a score ≥6 was assigned to tumors with high expression of DNA-PKcs. All slides were scanned digitally using a Bliss System (Bacus Laboratories/Olympus America, Lombard, IL, USA).

## Statistical analysis

IBM SPSS (version 25) and R statistical software were used for performing statistical analyses. Descriptive statistics were computed for continuous and categorical variables. The assessment of IHC expression scores against categorical groups was performed using the two-sided Wilcoxon rank-sum test for pair-wise comparisons and the Kruskal–Wallis rank-sum test for comparisons among more than two groups. Chi-square or Fisher exact tests were used to assess associations between DNA-PKcs expression and clinicopathological variables or expression of other biomarkers. Survival analysis was performed using breast cancer-specific survival (BCSS) as the prespecified primary endpoint, defined as the period between the date of diagnosis and the date of death attributed to breast cancer. Patients who were alive at the end of the follow-up period or who died due to causes other than breast cancer were censored. Cumulative survival probabilities were estimated by Kaplan-Meier methodology and differences in the survival rates between groups were calculated by log-rank testing. Cox proportional hazard modelling was used to compute univariate and multivariate analyses; hazard ratios with 95% confidence intervals were reported for each variable. Multivariate analysis was adjusted for clinicopathological variables including age at diagnosis, tumor size, grade, and nodal status. *P*-values of less than 0.05 were considered statistically significant.

### Bioinformatic analyses using publicly-available breast cancer datasets

The expression of *PRKDC* mRNA was assessed at the transcriptomic level using the TCGA cohort of breast invasive carcinomas^8^ and the Sweden Cancerome Analysis Network - Breast (SCAN-B) cohort^40^ (NCT02306096). TCGA data including *PRKDC* expression, PAM50 subtypes, reverse phase protein assay (RPPA) clusters, and IHC ER status were obtained through cBioPortal^74^. SCAN-B was accessed using the bc-GenExMiner v4.5 publicly-available tool^75^. Survival analyses for *PRKDC* mRNA expression were performed using the bc-GenExMiner v4.5 and the previously-established KMplotter analysis platform^41^ curated from 35 Gene Expression Omnibus datasets accessed using https://kmplot.com/analysis/. Kaplan–Meier survival curves were generated by partitioning cases according to the median mRNA expression.

### Analysis of PRKDC mutation data

Somatic mutations in a subset of 640 tamoxifen-treated, clinically ER + primary tumors from the BC Cancer series were available from a previous study that performed targeted sequencing of 83 biologically important genes including *PRKDC*^42^. Mutation lollipop diagrams were generated using the cBioPortal Mutation Mapper tool. Functional categorizations of *PRKDC* mutations were assessed using the “Mutation Assessor” with information using PolyPhen^76^ and SIFT^77^ tools.

### Reporting summary

Further information on research design is available in the Nature Research Reporting Summary linked to this article.

## Supplementary information

Supplementary Data 1

Supplementary Information

Reporting Summary

## Data Availability

An anonymized data file containing immunohistochemical data, molecular PAM50 subtype, and *PRKDC* mutation data used and analyzed in this study can be found in Supplementary Data 1. Clinical data for the patients included in this study are not publicly available per policy to protect patient privacy. Clinical data access can be made available to qualified researchers on a reasonable request through the Breast Cancer Outcomes Unit of BC Cancer, upon completion of a Data Transfer Agreement and confirmation of ethical approval. Reasonable requests or queries should be directed to the corresponding author. This study involved the collection and analysis of data from multiple publicly-available datasets. The TCGA breast cancer data analyzed can be accessed through the cBioPortal for Cancer Genomics repository (https://www.cbioportal.org/) - unique identifier: “Breast Invasive Carcinoma (TCGA, Nature 2021)”. SCAN-B was accessed using the bc-GenExMiner v4.5 publicly-available tool (http://bcgenex.centregauducheau.fr) – unique identifier: http://bcgenex.ico.unicancer.fr/BC-GEM/GEM-requete.php “RNA-seq/SCAN-B/*PRKDC*”. Survival analyses for *PRKDC* mRNA expression were performed using the bc-GenExMiner v4.5 and the previously-established KMplotter analysis platform accessed using (https://kmplot.com/analysis/) – unique identifier: https://kmplot.com/analysis/index.php?p=service&cancer=breast “*PRKDC*/Affymetrix_ID 208694_at”. *PRKDC* full genomic data and methods used for targeted sequencing can be found in Supplementary Data 3 of Griffith et al.^42^ (available online) – unique identifier: https://www.nature.com/articles/s41467-018-05914-x#Sec25 “Supplementary Data 3”.

## Code availability

No custom code was generated to collect data in this study. R codes used for analysis in this study are available from the corresponding author on a reasonable request.

## References

[1] Perou CM (2000). Molecular portraits of human breast tumours. Nature.

[2] Sørlie T (2001). Gene expression patterns of breast carcinomas distinguish tumor subclasses with clinical implications. Proc. Natl Acad. Sci. USA.

[3] Parker JS (2009). Supervised risk predictor of breast cancer based on intrinsic subtypes. J. Clin. Oncol..

[4] Robson M (2017). Olaparib for metastatic breast cancer in patients with a Germline BRCA mutation. N. Engl. J. Med..

[5] Schmid P (2018). Atezolizumab and nab-paclitaxel in advanced triple-negative breast cancer. N. Engl. J. Med..

[6] Garrido-Castro AC, Lin NU, Polyak K (2019). Insights into molecular classifications of triple-negative breast cancer: improving patient selection for treatment. Cancer Disco..

[7] Curtis C (2012). The genomic and transcriptomic architecture of 2,000 breast tumours reveals novel subgroups. Nature.

[8] Network CGA (2012). Comprehensive molecular portraits of human breast tumours. Nature.

[9] Lehmann BD (2011). Identification of human triple-negative breast cancer subtypes and preclinical models for selection of targeted therapies. J. Clin. Invest.

[10] Jiang YZ (2019). Genomic and transcriptomic landscape of triple-negative breast cancers: subtypes and treatment strategies. Cancer Cell.

[11] Burstein MD (2015). Comprehensive genomic analysis identifies novel subtypes and targets of triple-negative breast cancer. Clin. Cancer Res..

[12] Mertins P (2016). Proteogenomics connects somatic mutations to signalling in breast cancer. Nature.

[13] Krug K (2020). Proteogenomic landscape of breast cancer tumorigenesis and targeted therapy. Cell.

[14] Goodwin JF, Knudsen KE (2014). Beyond DNA repair: DNA-PK function in cancer. Cancer Disco..

[15] Yoo S, Dynan WS (1999). Geometry of a complex formed by double strand break repair proteins at a single DNA end: recruitment of DNA-PKcs induces inward translocation of Ku protein. Nucleic Acids Res..

[16] Shrivastav M (2009). DNA-PKcs and ATM co-regulate DNA double-strand break repair. DNA Repair.

[17] Chan DW (2002). Autophosphorylation of the DNA-dependent protein kinase catalytic subunit is required for rejoining of DNA double-strand breaks. Genes Dev..

[18] Lieber MR, Gu J, Lu H, Shimazaki N, Tsai AG (2010). Nonhomologous DNA end joining (NHEJ) and chromosomal translocations in humans. Subcell. Biochem..

[19] Sun G, Yang L, Dong C, Ma B, Shan M (2017). PRKDC regulates chemosensitivity and is a potential prognostic and predictive marker of response to adjuvant chemotherapy in breast cancer patients. Oncol. Rep..

[20] Cornell L (2015). DNA-PK-A candidate driver of hepatocarcinogenesis and tissue biomarker that predicts response to treatment and survival. Clin. Cancer Res..

[21] Li W, Xie C, Yang Z, Chen J, Lu NH (2013). Abnormal DNA-PKcs and Ku 70/80 expression may promote malignant pathological processes in gastric carcinoma. World J. Gastroenterol..

[22] Ciszewski WM, Tavecchio M, Dastych J, Curtin NJ (2014). DNA-PK inhibition by NU7441 sensitizes breast cancer cells to ionizing radiation and doxorubicin. Breast Cancer Res. Treat..

[23] Yang H, Yao F, Marti TM, Schmid RA, Peng RW (2020). Beyond DNA Repair: DNA-PKcs in tumor metastasis, metabolism and immunity. Cancers.

[24] Fok JHL (2019). AZD7648 is a potent and selective DNA-PK inhibitor that enhances radiation, chemotherapy and olaparib activity. Nat. Commun..

[25] Nik-Zainal S (2016). Landscape of somatic mutations in 560 breast cancer whole-genome sequences. Nature.

[26] Jeggo PA, Pearl LH, Carr AM (2016). DNA repair, genome stability and cancer: a historical perspective. Nat. Rev. Cancer.

[27] Nielsen TO (2004). Immunohistochemical and clinical characterization of the basal-like subtype of invasive breast carcinoma. Clin. Cancer Res..

[28] Chia SK, Speers CH, Bryce CJ, Hayes MM, Olivotto IA (2004). Ten-year outcomes in a population-based cohort of node-negative, lymphatic, and vascular invasion-negative early breast cancers without adjuvant systemic therapies. J. Clin. Oncol..

[29] Bortnik S (2016). Identification of breast cancer cell subtypes sensitive to ATG4B inhibition. Oncotarget.

[30] Lan T (2016). Targeting hyperactivated DNA-PKcs by KU0060648 inhibits glioma progression and enhances temozolomide therapy via suppression of AKT signaling. Oncotarget.

[31] Cheang MC (2008). Basal-like breast cancer defined by five biomarkers has superior prognostic value than triple-negative phenotype. Clin. Cancer Res..

[32] Loi S (2019). Tumor-infiltrating lymphocytes and prognosis: a pooled individual patient analysis of early-stage triple-negative breast cancers. J. Clin. Oncol..

[33] Park JH (2019). Prognostic value of tumor-infiltrating lymphocytes in patients with early-stage triple-negative breast cancers (TNBC) who did not receive adjuvant chemotherapy. Ann. Oncol..

[34] Won JR (2013). A survey of immunohistochemical biomarkers for basal-like breast cancer against a gene expression profile gold standard. Mod. Pathol..

[35] Asleh-Aburaya K (2016). Basal biomarkers Nestin and INPP4b accurately identify intrinsic subtype in breast cancers that are weakly positive for estrogen receptor. Histopathology.

[36] Prat A (2013). Molecular characterization of basal-like and non-basal-like triple-negative breast cancer. Oncologist.

[37] Deyarmin B (2013). Effect of ASCO/CAP guidelines for determining ER status on molecular subtype. Ann. Surg. Oncol..

[38] Iwamoto T (2012). Estrogen receptor (ER) mRNA and ER-related gene expression in breast cancers that are 1% to 10% ER-positive by immunohistochemistry. J. Clin. Oncol..

[39] Nielsen TO (2010). A comparison of PAM50 intrinsic subtyping with immunohistochemistry and clinical prognostic factors in tamoxifen-treated estrogen receptor-positive breast cancer. Clin. Cancer Res..

[40] Saal LH (2015). The Sweden Cancerome Analysis Network - Breast (SCAN-B) Initiative: a large-scale multicenter infrastructure towards implementation of breast cancer genomic analyses in the clinical routine. Genome Med..

[41] Györffy B (2010). An online survival analysis tool to rapidly assess the effect of 22,277 genes on breast cancer prognosis using microarray data of 1,809 patients. Breast Cancer Res. Treat..

[42] Griffith OL (2018). The prognostic effects of somatic mutations in ER-positive breast cancer. Nat. Commun..

[43] Altman DG, McShane LM, Sauerbrei W, Taube SE (2012). Reporting Recommendations for Tumor Marker Prognostic Studies (REMARK): explanation and elaboration. PLoS Med..

[44] Zhang Y (2019). High expression of PRKDC promotes breast cancer cell growth via p38 MAPK signaling and is associated with poor survival. Mol. Genet. Genom. Med..

[45] Anurag M (2018). Comprehensive Profiling of DNA Repair Defects in Breast Cancer Identifies a Novel Class of Endocrine Therapy Resistance Drivers. Clin. Cancer Res..

[46] Abdel-Fatah T (2014). Adverse prognostic and predictive significance of low DNA-dependent protein kinase catalytic subunit (DNA-PKcs) expression in early-stage breast cancers. Breast Cancer Res. Treat..

[47] Medunjanin S, Weinert S, Schmeisser A, Mayer D, Braun-Dullaeus RC (2010). Interaction of the double-strand break repair kinase DNA-PK and estrogen receptor-alpha. Mol. Biol. Cell.

[48] Arnold SF, Obourn JD, Jaffe H, Notides AC (1995). Phosphorylation of the human estrogen receptor by mitogen-activated protein kinase and casein kinase II: consequence on DNA binding. J. Steroid Biochem. Mol. Biol..

[49] Foulds CE (2013). Proteomic analysis of coregulators bound to ERα on DNA and nucleosomes reveals coregulator dynamics. Mol. Cell.

[50] Helzer KT (2019). The phosphorylated estrogen receptor α (ER) cistrome identifies a subset of active enhancers enriched for direct ER-DNA binding and the transcription factor GRHL2.. Mol. Cell Bio.l.

[51] Abdel-Fatah TM (2014). ATM, ATR and DNA-PKcs expressions correlate to adverse clinical outcomes in epithelial ovarian cancers. BBA Clin..

[52] Bolderson E, Richard DJ, Zhou BB, Khanna KK (2009). Recent advances in cancer therapy targeting proteins involved in DNA double-strand break repair. Clin. Cancer Res..

[53] O’Connor MJ (2015). Targeting the DNA damage response in cancer. Mol. Cell.

[54] Polo SE, Jackson SP (2011). Dynamics of DNA damage response proteins at DNA breaks: a focus on protein modifications. Genes Dev..

[55] Peng Y (2005). Deficiency in the catalytic subunit of DNA-dependent protein kinase causes down-regulation of ATM. Cancer Res..

[56] Tan KT (2020). PRKDC: new biomarker and drug target for checkpoint blockade immunotherapy.. J Immunother Cancer.

[57] Shintani S (2003). Up-regulation of DNA-dependent protein kinase correlates with radiation resistance in oral squamous cell carcinoma. Cancer Sci..

[58] Stronach EA (2011). DNA-PK mediates AKT activation and apoptosis inhibition in clinically acquired platinum resistance. Neoplasia.

[59] Salles B, Calsou P, Frit P, Muller C (2006). The DNA repair complex DNA-PK, a pharmacological target in cancer chemotherapy and radiotherapy. Pathol. Biol..

[60] Jackson SP, Bartek J (2009). The DNA-damage response in human biology and disease. Nature.

[61] Brown JS, Sundar R, Lopez J (2018). Combining DNA damaging therapeutics with immunotherapy: more haste, less speed. Br. J. Cancer.

[62] Dunn GP, Bruce AT, Ikeda H, Old LJ, Schreiber RD (2002). Cancer immunoediting: from immunosurveillance to tumor escape. Nat. Immunol..

[63] Farmer H (2005). Targeting the DNA repair defect in BRCA mutant cells as a therapeutic strategy. Nature.

[64] Eikesdal HP (2021). Olaparib monotherapy as primary treatment in unselected triple negative breast cancer. Ann. Oncol..

[65] Chopra N (2020). Homologous recombination DNA repair deficiency and PARP inhibition activity in primary triple negative breast cancer. Nat. Commun..

[66] Patel AG, Sarkaria JN, Kaufmann SH (2011). Nonhomologous end joining drives poly(ADP-ribose) polymerase (PARP) inhibitor lethality in homologous recombination-deficient cells. Proc. Natl Acad. Sci. USA.

[67] Strickland KC (2016). Association and prognostic significance of BRCA1/2-mutation status with neoantigen load, number of tumor-infiltrating lymphocytes and expression of PD-1/PD-L1 in high grade serous ovarian cancer. Oncotarget.

[68] Chatzinikolaou G, Karakasilioti I, Garinis GA (2014). DNA damage and innate immunity: links and trade-offs. Trends Immunol..

[69] Cheang MC (2006). Immunohistochemical detection using the new rabbit monoclonal antibody SP1 of estrogen receptor in breast cancer is superior to mouse monoclonal antibody 1D5 in predicting survival. J. Clin. Oncol..

[70] Salgado R (2015). The evaluation of tumor-infiltrating lymphocytes (TILs) in breast cancer: recommendations by an International TILs Working Group 2014. Ann. Oncol..

[71] Burugu S, Gao D, Leung S, Chia SK, Nielsen TO (2018). TIM-3 expression in breast cancer. Oncoimmunology.

[72] Liu S (2012). CD8+ lymphocyte infiltration is an independent favorable prognostic indicator in basal-like breast cancer. Breast Cancer Res..

[73] Liu S (2017). Role of cytotoxic tumor-infiltrating lymphocytes in predicting outcomes in metastatic HER2-positive breast cancer: a secondary analysis of a randomized clinical trial. JAMA Oncol..

[74] Gao J (2013). Integrative analysis of complex cancer genomics and clinical profiles using the cBioPortal. Sci. Signal.

[75] Jézéquel P (2012). bc-GenExMiner: an easy-to-use online platform for gene prognostic analyses in breast cancer. Breast Cancer Res. Treat..

[76] Ramensky V, Bork P, Sunyaev S (2002). Human non-synonymous SNPs: server and survey. Nucleic Acids Res..

[77] Kumar P, Henikoff S, Ng PC (2009). Predicting the effects of coding non-synonymous variants on protein function using the SIFT algorithm. Nat. Protoc..

