# Supplementary material for: Proteomics-derived basal biomarker DNA-PKcs is associated with intrinsic subtype and long-term clinical outcomes in breast cancer
Source: NPJ Breast Cancer. 2021 Sep 9;7:114. doi: 10.1038/s41523-021-00320-x (PMC8429676; doi:10.1038/s41523-021-00320-x)
Supplement: Supplementary file 2 — Supplementary Information [file 41523_2021_320_MOESM2_ESM.pdf]

Supplementary Information

Proteomics-derived basal biomarker DNA-PKcs is associated with intrinsic subtype and long-term clinical outcomes in breast cancer

Karama Asleh, Nazia Riaz, Angela S. Cheng, Dongxia Gao, Samuel C. Y. Leung, Meenakshi Anurag, Torsten O. Nielsen

Supplementary Information includes:

1. Supplementary Figures (page 2-6)
2. Supplementary Table (pages 7-8)
3. Caption for Supplementary Data 1 (page 9)

Supplementary Figure 1

a

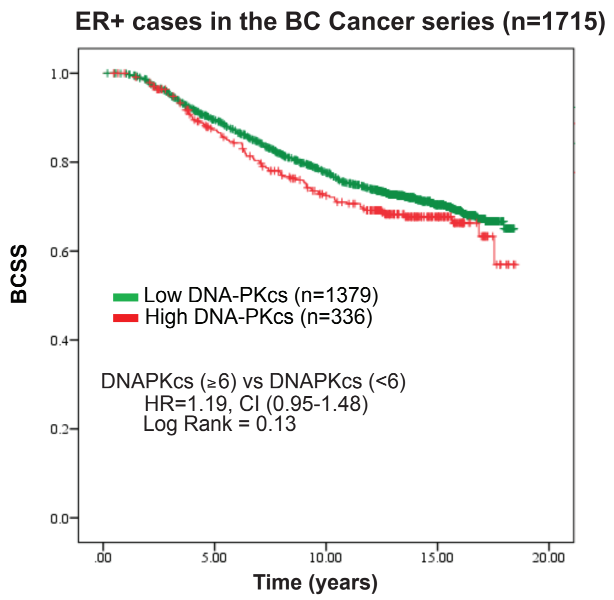

b

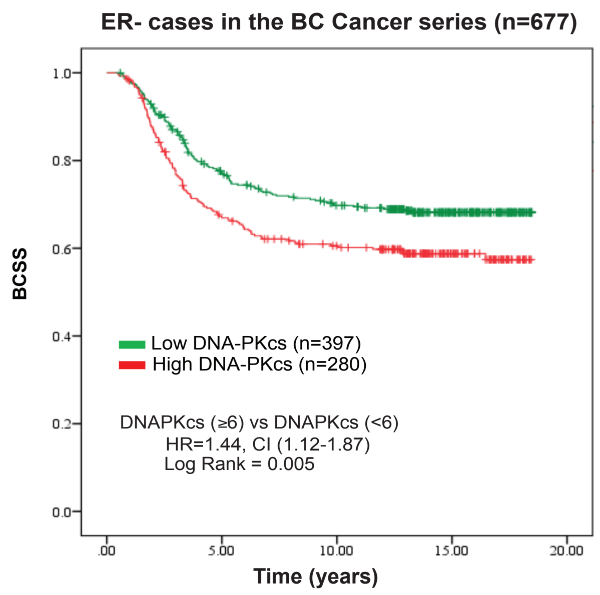

Supplementary Fig. 1: Prognostic significance of DNA-PKcs expression by immunohistochemistry in different ER subgroups of the BC Cancer series. Kaplan Meier

curves for BCSS in ER+ cases (a), and ER- cases (b). Abbreviations: BCSS, breast cancer specific survival; ER, estrogen receptor.

Supplementary Figure 2

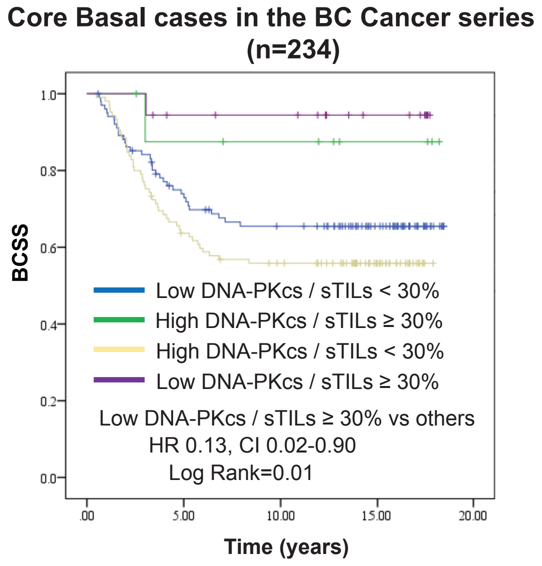

Supplementary Fig. 2: Prognostic stratification of core basal cases by DNA-PKcs and stromal TILs in the BC Cancer series using a higher (30%) cutpoint. Within core basal tumors, presence of sTILs (≥30% vs < 30%) in combination with low DNA-PKcs expression is associated with better BCSS. Abbreviations: BCSS, breast cancer specific survival; HR, hazard ratio, TIL, tumor infiltrating lymphocytes.

Supplementary Figure 3

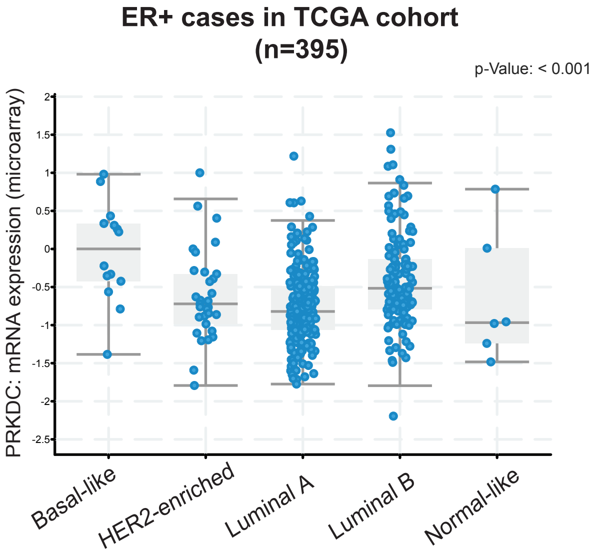

Supplementary Fig. 3: Boxplots showing that expression levels of *PRKDC,* as derived from the 395 ER+ breast cancers in TCGA are significantly associated with basal-like PAM50 intrinsic subtype. Centre bar denotes median, and the upper and lower box boundaries delineate the third and first quartiles.

Supplementary Figure 4

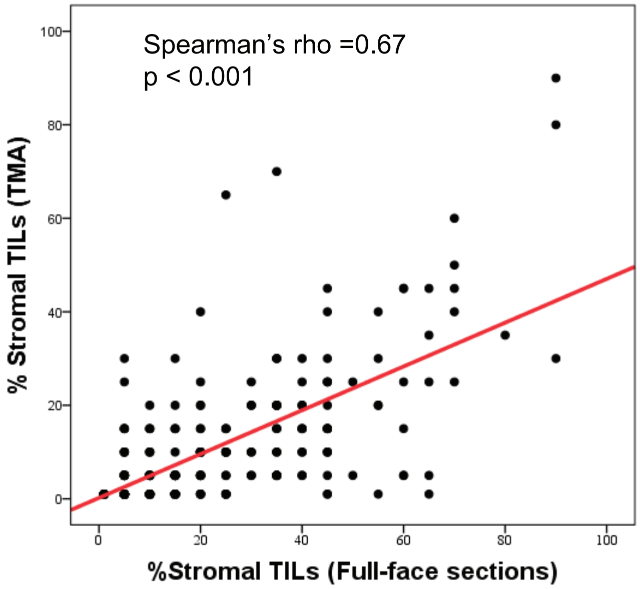

Supplementary Fig. 4: Scatter plot showing correlation between morphologically assessed stromal TILs on full-face sections and tissue microarrays in BC Cancer series (n=317). Each data point represents one case. The Spearman correlation coefficient (*rho*) and p-values are displayed. Abbreviations: TILs, tumor infiltrating lymphocytes; TMA, tissue microarray.

Supplementary Tables:

Supplementary Table 1: Association of DNA-PKcs expression with clinicopathological features in the UBC series

Clinicopathological Features	DNA-PKcs Expression (IHC score)		*P*-value
	Low (< 6) n = 233	High (≥ 6) n = 67	
Age at diagnosis			0.51
< 50	87 (37.3)	28 (41.8)	
≥ 50	146 (62.7)	39 (58.2)	
Tumor size (cm)			0.97
≤ 2 cm	135 (57.9)	39 (58.2)	
>2 cm	98 (42.1)	28 (41.8)	
Tumor grade			<0.001
Grade 1 or 2	145 (63)	22 (32.8)	
Grade 3	85 (37)	45 (67.2)	
Axillary lymph node status			0.10
Positive	76 (35.5)	31 (48.4)	
Negative	138 (64.5)	33 (51.6)	
Lymphovascular invasion			0.01
Positive	46 (20.6)	24 (35.8)	
Negative	177 (79.4)	43 (64.2)	
Ki-67 (%)			<0.001
< 14	147 (64.5)	24 (36.4)	
≥ 14%	81 (35.5)	42 (63.6)	
ER expression			0.007
Negative	41 (17.8)	22 (33.3)	
Positive	189 (82.2)	44 (66.7)	
Progesterone receptor expression			0.06
Negative	69 (30.1)	28 (42.4)	
Positive	160 (69.9)	38 (57.6)	
HER2 expression			0.14
Negative	217 (95.6)	54 (87.1)	
Positive	10 (4.4)	8 (12.9)	
EGFR expression			0.004
Negative	211 (91.3)	51 (78.5)	
Positive	20 (8.7)	14 (21.5)	

CK5/6 expression			0.05
Negative	217 (94.8)	57 (87.7)	
Positive	12 (5.2)	8 (12.3)	
IHC subtype			<0.001
Luminal A ([ER+ or PR+], HER2-, low Ki67)	132 (59.2)	18 (29.5)	
Luminal B ([ER+ or PR+], HER2-, high Ki67)	51 (22.9)	20 (32.8)	
Luminal B ([ER+ or PR+], HER2+)	5 (2.2)	2 (3.3)	
ER-, PR-, HER2+	5 (2.2)	6 (9.8)	
ER-, PR-, HER2-	30 (13.5)	15 (24.6)	
Triple negative phenotype			0.03
Yes	30 (13.5)	15 (24.6)	
No	193 (86.5)	46 (75.4)	
Core basal subtype			0.02
Yes	17 (7.3)	11 (16.4)	
No	216 (92.7)	56 (83.6)	
Stromal TILs (H&E)			0.11
< 10%	191 (82)	49 (73.1)	
≥ 10%	42 (18)	18 (26.9)	
CD8 iTIL count			0.006
< 1	93 (49.2)	14 (27.5)	
≥ 1	96 (50.8)	37 (72.5)	

Abbreviations: IHC, immunohistochemistry; ER, estrogen receptor; PR, progesterone receptor; HER2, human epidermal growth factor receptor 2; EGFR, epidermal growth factor receptor; CK, cytokeratin; H&E, hematoxylin and eosin; CD, cluster of differentiation.

Supplementary Data 1 (appears as an Excel file): Characteristics of the BC Cancer series including pathological features for the whole cohort and genomic *PRKDC* mutation data for the ER+ subset.
