# Supplementary material for: Proteomics-derived basal biomarker DNA-PKcs is associated with intrinsic subtype and long-term clinical outcomes in breast cancer
Source: NPJ Breast Cancer. 2021 Sep 9;7:114. doi: 10.1038/s41523-021-00320-x (PMC8429676; doi:10.1038/s41523-021-00320-x)
Supplement: Supplementary file 3 — Reporting Summary [file 41523_2021_320_MOESM3_ESM.pdf]

nature portfolio

Corresponding author(s): TORSTEN O. NIELSEN

Last updated by author(s): Jun 7, 2021

Reporting Summary

Nature Portfolio wishes to improve the reproducibility of the work that we publish. This form provides structure for consistency and transparency in reporting. For further information on Nature Portfolio policies, see our Editorial Policies and the Editorial Policy Checklist.

Statistics

For all statistical analyses, confirm that the following items are present in the figure legend, table legend, main text, or Methods section.

n/a	Confirmed	
☐	☒	The exact sample size (n) for each experimental group/condition, given as a discrete number and unit of measurement
☐	☒	A statement on whether measurements were taken from distinct samples or whether the same sample was measured repeatedly
☐	☒	The statistical test(s) used AND whether they are one- or two-sided<br>*Only common tests should be described solely by name; describe more complex techniques in the Methods section.*
☐	☒	A description of all covariates tested
☐	☒	A description of any assumptions or corrections, such as tests of normality and adjustment for multiple comparisons
☐	☒	A full description of the statistical parameters including central tendency (e.g. means) or other basic estimates (e.g. regression coefficient) AND variation (e.g. standard deviation) or associated estimates of uncertainty (e.g. confidence intervals)
☐	☒	For null hypothesis testing, the test statistic (e.g. F, t, r) with confidence intervals, effect sizes, degrees of freedom and P value noted<br>*Give P values as exact values whenever suitable.*
☒	☐	For Bayesian analysis, information on the choice of priors and Markov chain Monte Carlo settings
☐	☒	For hierarchical and complex designs, identification of the appropriate level for tests and full reporting of outcomes
☐	☒	Estimates of effect sizes (e.g. Cohen's d, Pearson's r), indicating how they were calculated

Our web collection on statistics for biologists contains articles on many of the points above.

Software and code

Policy information about availability of computer code

Data collection	No custom code was used to collect data in this study.
Data analysis	R Statistical Computing Software, The R Environment http://www.r-project.org<br>IBM SPSS (version 25)<br><br>cBioPortal for Cancer Genomics repository (https://www.cbioportal.org/)<br>bc-GenExMiner v4.5 publicly-available tool (http://bcgenex.centregauducheau.fr)<br>KMplotter analysis platform (https://kmplot.com/analysis/)

For manuscripts utilizing custom algorithms or software that are central to the research but not yet described in published literature, software must be made available to editors and reviewers. We strongly encourage code deposition in a community repository (e.g. GitHub). See the Nature Portfolio guidelines for submitting code & software for further information.

Data

Policy information about availability of data

All manuscripts must include a data availability statement. This statement should provide the following information, where applicable:

- Accession codes, unique identifiers, or web links for publicly available datasets
- A description of any restrictions on data availability
- For clinical datasets or third party data, please ensure that the statement adheres to our policy

Immunohistochemical data, molecular PAM50 subtype and PRKDC mutation data used in this study can be found in Supplementary Table 2. Clinical data for the TMAs used in this study can be made available to qualified researchers through the Breast Cancer Outcomes Unit of BC Cancer, upon completion of a Data Transfer Agreement and confirmation of ethical approval. The TCGA breast cancer data analyzed can be accessed through the cBioPortal for Cancer Genomics repository (https://www.cbioportal.org/). SCAN-B was accessed using the bc-GenExMiner v4.5 publicly-available tool (http://bcgenex.centregauducheau.fr). Survival analyses for PRKDC mRNA expression were performed using the bc-GenExMiner v4.5 and the previously-established KMplotter analysis platform accessed using (https://kmplot.com/analysis/). PRKDC full genomic data and methods used for targeted sequencing have been previously published and can be found in Supplementary Table 3 of Griffith et al (available online).

Field-specific reporting

Please select the one below that is the best fit for your research. If you are not sure, read the appropriate sections before making your selection.

☒ Life sciences ☐ Behavioural & social sciences ☐ Ecological, evolutionary & environmental sciences

For a reference copy of the document with all sections, see nature.com/documents/nr-reporting-summary-flat.pdf

Life sciences study design

All studies must disclose on these points even when the disclosure is negative.

Sample size	Sample size of 300 tumor samples were included in the study as a training set and 2401 as a validation set. This number is enough powered to allow performing meaningful clinical correlations for biomarker analyses
Data exclusions	Patients diagnosed with ductal carcinoma in situ only, metastatic disease at presentation, and those who received neoadjuvant therapies were excluded from the analysis. Cases on tissue microarrays that were uninterpretable by immunohistochemistry were excluded from the analysis.
Replication	Duplicate 0.6 mm cores were assessed on tissue microarrays and these were originally extracted from each patient's pathology block
Randomization	N/A as clinical materials were not obtained by a randomization design followed for this specific study
Blinding	N/A as there were no experimental interventions to which investigators could be blinded

Reporting for specific materials, systems and methods

We require information from authors about some types of materials, experimental systems and methods used in many studies. Here, indicate whether each material, system or method listed is relevant to your study. If you are not sure if a list item applies to your research, read the appropriate section before selecting a response.

Materials & experimental systems

n/a	Involved in the study	
☐	☒	Antibodies
☒	☐	Eukaryotic cell lines
☒	☐	Palaeontology and archaeology
☒	☐	Animals and other organisms
☐	☒	Human research participants
☐	☒	Clinical data
☒	☐	Dual use research of concern

Methods

n/a	Involved in the study	
☒	☐	ChIP-seq
☒	☐	Flow cytometry
☒	☐	MRI-based neuroimaging

Antibodies

Antibodies used	anti DNA-PKcs rabbit monoclonal antibody (clone Y393, Abcam, cat# ab32566).<br>anti-CD8 monoclonal mouse antibody (clone [C8/144B], Dako, cat# M7103)
Validation	Array sections at 4µm were mounted on glass slides and baked for an hour at 60°C to prepare for staining on a Ventana Discovery XT

Validation	automated stainer (Ventana Medical Systems, Tucson, AZ). Antigen retrieval was performed using Cell Conditioning 1 antigen retrieval (Ventana Medical Systems) followed by 2 hours of primary antibody incubation at room temperature, and detected using a ChromoMap DAB Detection Kit (Ventana Medical Systems). IHC staining of DNA-PKcs was performed with anti DNA-PKcs rabbit monoclonal primary antibody (clone Y393, dilution 1:500, Abcam, cat# ab32566). Slides were then incubated with a secondary antibody (UltraMap anti-Rb HRP) for an additional 16 minutes.

Human research participants

Policy information about studies involving human research participants

Population characteristics	The staining protocol, scoring criteria and clinical data analysis were first evaluated on a set of female patients diagnosed with invasive breast cancer (n=330) at the University of British Columbia (UBC) hospital between 1998-2002, designated as the UBC series. The second cohort was used for subsequent detailed analyses and is comprised of primary invasive breast cancer cases diagnosed in the province of British Columbia at the British Columbia Cancer Agency between 1986-1992, referred to as the BC Cancer series. These patients were treated in accordance with the provincial guidelines during the specified time-period.
Recruitment	No patients were recruited as part of this study.
Ethics oversight	This study was approved by the research ethics board of the University of British Columbia and the BC Cancer Breast Cancer Outcomes unit (approval number: H17-01207). Consent for the use of previously assembled patient specimens was obtained under waiver of informed consent policy without identification of patient information.

Note that full information on the approval of the study protocol must also be provided in the manuscript.

Clinical data

Policy information about clinical studies

All manuscripts should comply with the ICMJE guidelines for publication of clinical research and a completed CONSORT checklist must be included with all submissions.

Clinical trial registration	N/A
Study protocol	N/A
Data collection	This study used clinical data collected and updated periodically by the BC Cancer Breast Cancer Outcome Unit, University of British Columbia Canada
Outcomes	Breast cancer specific survival (BCSS) was used as the prespecified primary endpoint, defined as the period between the date of diagnosis and the date of death attributed to breast cancer. Patients who were alive at the end of the follow-up period or who died due to causes other than breast cancer were censored. Cumulative survival probabilities were estimated by Kaplan-Meier methodology and differences in the survival rates between groups calculated by log-rank testing. Cox proportional hazard modelling was used to compute univariate and multivariate analyses; hazard ratios with 95% confidence intervals was reported for each variable.
